# OA09.04. NEonatology and Osteopathy (NEO) Study: effect of OMT on preterms’ length of stay

**DOI:** 10.1186/1472-6882-12-S1-O36

**Published:** 2012-06-12

**Authors:** F Cerritelli, G Pizzolorusso, F Ciardelli, E La Mola, C Renzetti, V Cozzolino, G Barlafante

**Affiliations:** 1 European Institute for Evidence Based Osteopathic Medicine, Pescara, Italy; 2 Accademia Italiana Osteopatia Tradizionale, Pescara, Italy

## Purpose

The use of osteopathic manipulative treatment (OMT) in preterm infants has been documented and results from previous studies suggest the association between OMT and length of stay (LOS) reduction, as well as significant improvement in several clinical outcomes. The aim of the present study is to show the effect of OMT on LOS and daily weight gain in a sample of premature infants.

## Methods

Randomized controlled trial on preterm newborns admitted in a single NICU between 2008-2009. N=101 subjects free of medical complications and with gestational age >28 and < 38 weeks were enrolled and randomized into two groups: study group (N=47) and control group (N=54). All subjects received routine pediatric care and OMT was performed to the study group for the entire period of hospitalization. Endpoints of the study included differences in LOS and daily weight gain. Statistical analyses were based on univariate tests and multivariate linear regression.

## Results

Univariate statistical analysis showed no significant imbalances among treated and control groups in terms of main characteristics measured at admission. At the end of follow-up, OMT was significantly associated with LOS (days) [27.3±17.3 vs 31.5±21.7, p=0.03] and with daily weight gain (grams) [65.1±28.5 vs 58.6±28.8, p=0.03]. After adjusting for all potential confounders, multivariate analysis showed a significant association between OMT and LOS reduction (mean difference between treated and control group: -6.325; 95% CI -8.687, -3.962; p< 0.0001). OMT was not independently associated with any change in daily weight gain.

## Conclusion

The present study suggests that OMT plays an important role in the management of preterm infants hospitalization.

